# Atlanta Medical College Museum

**Published:** 1859-02

**Authors:** 


### ATLANTA MEDICAL COLLEGE MUSEUM.

We desire to inform those who are feeling an interest in our prosperity, that the Faculty of the Atlanta Medical College, have succeeded in securing the services of a competent gentleman, who was appointed Curator of the Museum, some months ago.

By a recent arrangement, his entire time will be devoted to that department, which will constantly increase in value and interest, under his skilful management.

